# NAHA, a Novel Hydroxamic Acid-Derivative, Inhibits Growth and Angiogenesis of Breast Cancer *In Vitro* and *In Vivo*

**DOI:** 10.1371/journal.pone.0034283

**Published:** 2012-03-29

**Authors:** Jiahua Jiang, Anita Thyagarajan-Sahu, Viktor Krchňák, Andrej Jedinak, George E. Sandusky, Daniel Sliva

**Affiliations:** 1 Cancer Research Laboratory, Methodist Research Institute, Indiana University Health, Indianapolis, Indiana, United States of America; 2 Department of Chemistry and Biochemistry, University of Notre Dame, Notre Dame, Indiana, United States of America; 3 Department of Pathology, School of Medicine, Indiana University, Indianapolis, Indiana, United States of America; 4 Department of Medicine, School of Medicine, Indiana University, Indianapolis, Indiana, United States of America; 5 IU Simon Cancer Center, School of Medicine, Indiana University, Indianapolis, Indiana, United States of America; Wayne State University School of Medicine, United States of America

## Abstract

**Background:**

We have recently synthesized novel *N*-alkylated amino acid-derived hydroxamate, 2-[Benzyl-(2-nitro-benzenesulfonyl)-amino]-N-hydroxy-3-methyl-N-propyl-butyramide (NAHA). Here, we evaluate the anticancer activity of NAHA against highly invasive human breast cancer cells MDA-MB-231 *in vitro* and *in vivo*.

**Methodology/Principal Findings:**

Cell growth was evaluated by MTT and soft agar assays. Protein expression was determined by DNA microarray and Western blot analysis. Metastatic potential was evaluated by cell adhesion, migration, invasion, capillary morphogenesis, and ELISA assays. The anticancer activity *in vivo* was evaluated in mouse xenograft model. NAHA inhibited proliferation and colony formation of MDA-MB-231 cells together with the down-regulation of expression of Cdk2 and CDC20 proteins. NAHA inhibited cell adhesion, migration, and invasion through the suppression of secretion of uPA. NAHA suppressed secretion of VEGF from MDA-MB-231 cells and inhibited capillary morphogenesis of human aortic endothelial cells (HAECs). Finally, NAHA at 50 mg/kg was not toxic and decreased tumor volume and tumor weight *in vivo*. This suppression of tumor growth was associated with the inhibition of mitotic figures and induction of apoptosis, and the reduction of CD31 and VEGF positive cells in tumors.

**Conclusion:**

NAHA could be a novel promising compound for the development of new drugs for the therapy of invasive breast cancers.

## Introduction

Hydroxamic acids are able to chelate metal ions and therefore inhibit metal-containing enzymes such as matrix metalloproteinases (MMPs) [1]. Overexpression of MMPs has been linked to a variety of diseases including cancer, rheumatoid arthritis, osteoarthritis and cardiovascular disease [2], [3]. Despite the promising preclinical studies with synthetic hydroxamate-based MMP inhibitors, these inhibitors were not specific only for MMPs and most importantly, clinical studies revealed severe adverse side-effect such as the musculoskeletal syndrome [3], [4]. Therefore, the development of selectively specific MMP inhibitors without their side effects is necessary for the clinical studies [5]. However, certain chemical modifications in the structure of hydroxamic acids, such as *N*-alkylation, eliminated their MMP inhibitory activity [6], [7], [8]. We have recently synthesized a small library of *N*-alkylated amino acid-derived sulfonamide hydroxamates, and identified *2-[Benzyl-(2-nitro-benzenesulfonyl)-amino]-N-hydroxy-3-methyl-N-propyl-butyramide* (NAHA) as the most potent inhibitor of proliferation of highly invasive human breast cancer cells [9].

Breast cancer is the leading cause of cancer death in women worldwide [10]. One of the reasons for such a high mortality is invasive behavior of breast cancer cells. Therefore, breast cancer often progresses from the nonmetastatic and therapy-responsive phenotype to the highly invasive and metastatic phenotype, which is usually resistant to standard therapeutic procedures [11], [12]. Cancer metastasis consist from several interdependent processes including uncontrolled growth of cancer cells, their invasion through surrounding tissues, migration to the distant sites of the human body, and adhesion, invasion and colonization of other organs and tissues [13]. In addition, tumor growth and metastasis also require angiogenesis, the formation of blood vessels by capillaries sprouting from pre-existing vessels [14]. Therefore, suppression of growth and invasiveness of cancer cells, and cancer cells mediated angiogenesis could lead to the inhibition of cancer metastasis and would eventually further increase survival of breast cancer patients.

In the present study, we evaluated the effect of NAHA on highly invasive MDA-MB-231 cells representing metastatic human breast cancer cells. Here, we show that NAHA inhibits cell proliferation (anchorage-dependent growth) as well as colony formation (anchorage-independent cell growth) of MDA-MB-231 cells. In addition, NAHA inhibits invasive behavior (cell adhesion, migration and invasion) of breast cancer cells, and suppresses breast cancer cell-mediated angiogenesis of vascular endothelial cells *in vitro*. Finally, NAHA inhibits tumor growth and angiogenesis in a xenograft model of breast cancer. Collectively, our study suggests newly synthesized derivative of hydroxamic acid (NAHA) for the treatment of breast cancer.

## Results

### Effect of NAHA on the proliferation and colony formation of highly invasive breast cancer cells

**Figure 1:**
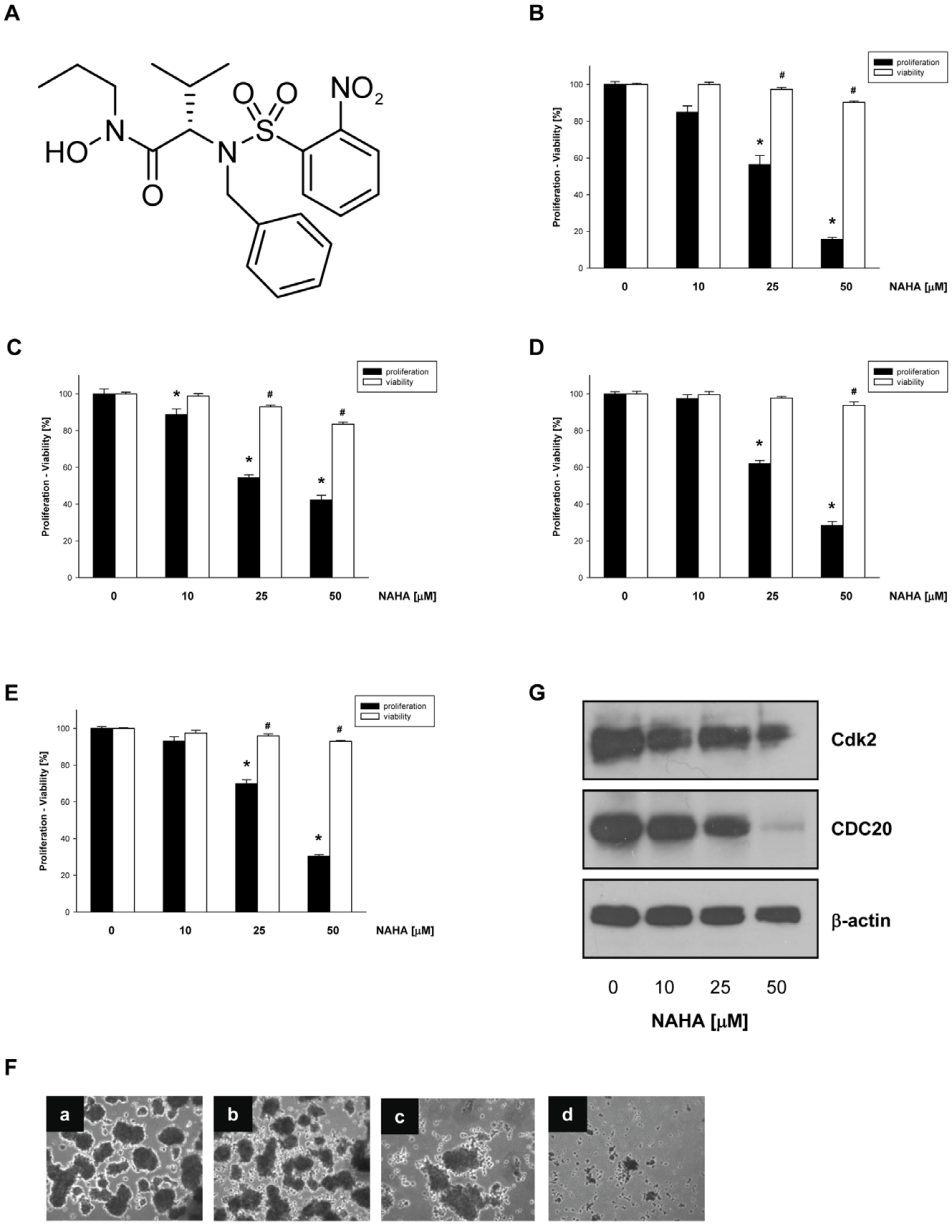
NAHA inhibits growth of breast cancer cells. (A) Structure of NAHA, *2-[Benzyl-(2-nitro-benzenesulfonyl)-amino]-N-hydroxy-3-methyl-N-propyl-butyramide*. (B) MDA-MB-231, (C) MCF-7, (D) MCF-10A, (E) HMEC cells were treated with NAHA (0–50 µM). Cell proliferation was determined by the tetrazolium salt method as described in *Materials and Methods*. Cell viability was determined by trypan blue staining as described in *Materials and Methods*. Data are the means ± SD. Similar results were obtained in at least two additional experiments. * p<0.05 for cell proliferation, # p<0.05 for cell viability. (F) Anchorage-independent growth (colony formation) of MDA-MB-231 cells was assessed on 1% agarose after incubation with NAHA (a – 0, b – 10 µM, c -25 µM, d – 50 µM) for 14 days as described in *Materials and Methods*. (G) MDA-MB-231 cells were treated with NAHA (0–50 µM) for 24 hours and whole cell extracts were subjected to Western blot analysis with anti-Cdk2 and anti-CDC20 antibodies as described in *Materials and Methods*. The equal protein loading was verified with anti β-actin antibodies. The rfesults are representative of three independent experiments.

We have recently synthesized and identified *N*-alkylated amino acid-derived sulfonamide hydroxamate, NAHA (Figure 1A), which inhibited growth of human breast cancer cells at IC_50_ 30 µM [9]. To compare the effect of NAHA on highly invasive (MDA-MB-231), poorly invasive (MCF-7) breast cancer cells, and human mammary epithelial cells (MCF-10A) and primary human mammary epithelial cells (HMECs) we treated these cells with NAHA (0–50 µM) for 24 hours and the proliferation and cell viability were determined. Interestingly, NAHA inhibited proliferation of all tested cells but the inhibition of cell proliferation was not associated with cell viability (Figure 1B–E). Therefore, NAHA at 50 µM suppressed proliferation of MDA-MB-231 by 84% (Figure 1B), MCF-7 by 58% (Figure 1C), MCF-10A by 72% (Figure 1D) and HMECs by 70% (Figure 1E) suggesting that the most sensitive to NAHA are highly invasive breast cancer cells MDA-MB-231.

Cancer cells can growth under the nonadhesive condition and this anchorage-independent growth (colony formation) is correlated with *in vivo* oncogenic potential of cancer cells. Because colony formation is a key parameter for cells to acquire a metastatic potential [15], we evaluated effects of NAHA on colony formation of highly invasive MDA-MB-231 cells. In agreement with cell proliferation, NAHA decreased the number of colonies of MDA-MB-231 cells (Figure 1F). These results suggest that NAHA inhibited the anchorage-dependent (cell proliferation) as well as anchorage-independent (colony formation) growth of invasive breast cancer cells.

### Effect of NAHA on the expression of cell cycle regulatory proteins

Since NAHA suppressed growth of breast cancer cells, we were interested which of the cell cycle regulatory proteins could be potential targets for this compound. MDA-MB-231 cells were treated with NAHA (0, 25, 50 µM) for 24 hours, RNA was extracted and the expression of cell cycle regulatory genes was evaluated by Cycle Oligo GEArray. Our data demonstrate that NAHA at the concentration 50 µM markedly down-regulated mRNA levels of several genes, including *ANAPC4* (ratio to control 0.53), *ANAPC5* (0.58), *cyclin B1* (0.61), *cyclin H* (0.53), *CDC20* (0.68), *CDC25C* (0.80), *Cdk2* (0.70), *Ki 67* (0.55), *CKS1B* (0.57), *CKS2* (0.65), *culin 1* (0.47), *E2F1* (0.70), *MCM2* (0.66), *TFDP1* (0.68), and *survivin* (0.82) in MDA-MB-231 cells. To confirm that NAHA regulates expression of these genes on the protein level, MDA-MB-231 cells were treated with NAHA (0–50 µM) for 24 hours, whole cell extracts prepared and subjected to Western blot analysis. Although the expression of some proteins, e.g. cyclin-B1 or PCNA, was down-regulated only marginally (not shown), NAHA markedly suppressed expression of cyclin-dependent kinase 2 (Cdk2) and cell division cycle 20 (CDC20) proteins, respectively (Figure 1G).

### Effect of NAHA on invasive behavior of breast cancer cells

**Figure 2:**
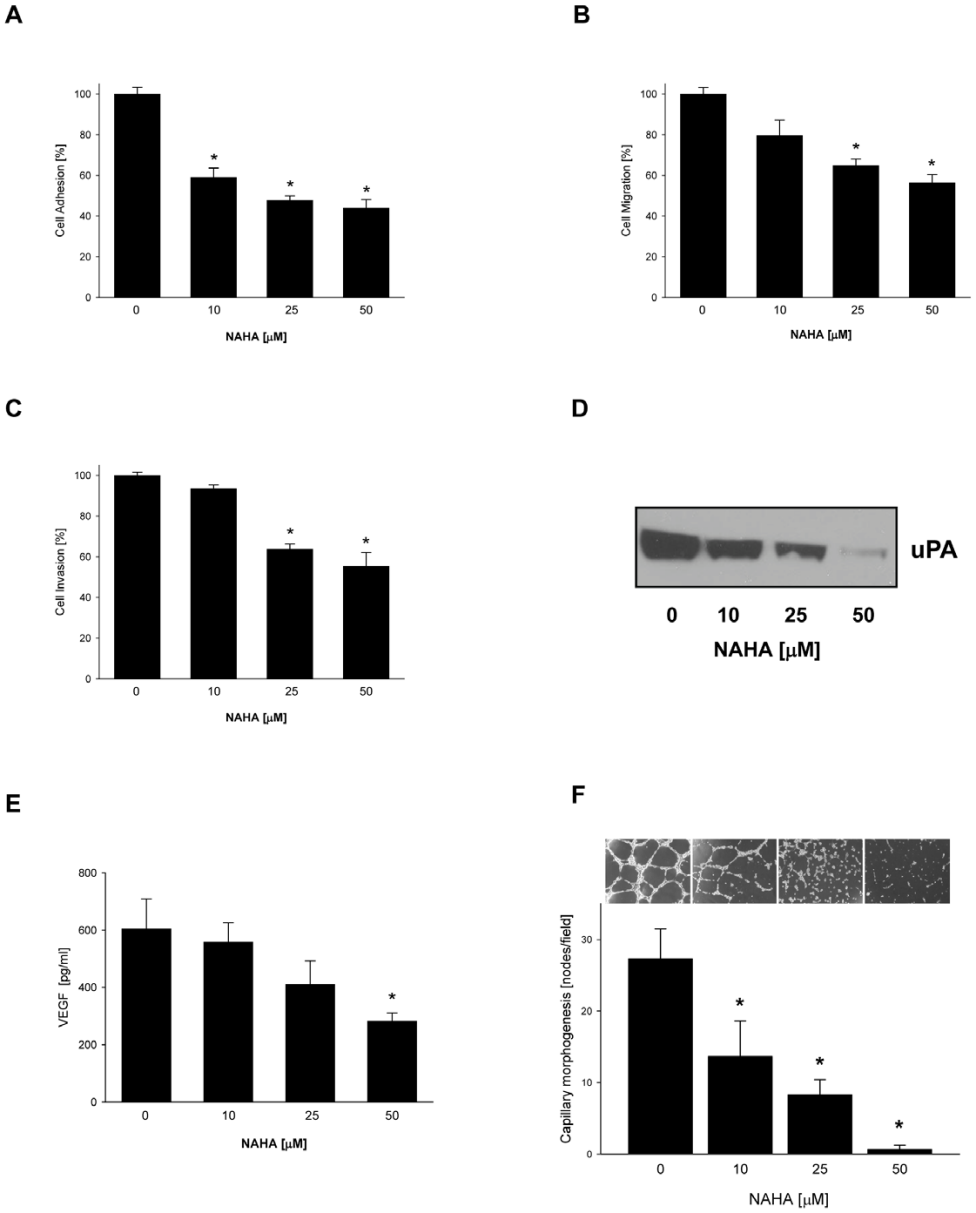
NAHA inhibits invasive behavior of breast cancer cells and capillary morphogenesis of endothelial cells. (A) Cell adhesion. MDA-MB-231 cells were treated with NAHA (0–50 µM) for 24 hours and cell adhesion to vitronectin determined as described in *Materials and Methods*. Each bar represents the mean ± SD of three experiments. * p<0.05. (B) Cell migration. Cell migration of MDA-MB-231 cells was determined after 5 hours of incubation in the presence of NAHA (0–50 µM), as described in *Materials and Methods*. Each bar represents the mean ± SD of three experiments. * p<0.05. (C) Cell invasion. Invasion of MDA-MB-231 cells through Matrigel was determined after 24 hours of incubation in the presence of NAHA (0–50 µM) as described in *Materials and Methods*. Each bar represents the mean ± SD of three experiments. * p<0.05. (D) uPA secretion. MDA-MB-231 cells were treated with NAHA (0–50 µM) for 24 hours, and the expression of uPA detected in conditioned media from the same amount of cells with anti-uPA antibody by Western blot analysis as described in *Materials and Methods*. The results are representative of three independent experiments. (E) MDA-MB-231 cells were treated with NAHA (0–50 µM) for 24 hours, media collected and secretion of VEGF determined as described in *Materials and Methods*. Each bar represents the mean ± SD of three experiments. * p<0.05. (F) HAECs were treated with NAHA (0–50 µM) for 16 hours. Capillary morphogenesis was determined as described in *Materials and Methods*. Each bar represents the mean ± SD of three experiments. * p<0.05.

In addition to the uncontrolled proliferation and colony formation, cancer metastasis depends on adhesion, migration and invasion of cancer cells. Breast cancer cells express integrin receptor α_V_β_3_, which through its interaction with an extracellular matrix (ECM) protein vitronectin, contributes to the cancer cell adhesion and migration [16]. To evaluate whether NAHA inhibits adhesion of breast cancer cells, MDA-MB-231 cells were treated with NAHA (0–50 µM) for 24 hours and their adhesion to vitronectin was determined. As seen in Figure 2A, NAHA markedly suppressed adhesion of MDA-MB-231 cells to extracellular matrix protein vitronectin. Further, we evaluated if NAHA directly inhibits cell migration. MDA-MB-231 cells were pretreated with NAHA (0–50 µM) for 1 h and cell migration was determined after additional 4 hours of incubation. As seen in Figure 2B, NAHA also significantly decreased migratory potential of breast cancer cells in a dose dependent-manner. Finally, we evaluated the effect of NAHA on cell invasiveness. MDA-MB-231 cells were plated on the Matrigel-coated filters in the presence of NAHA (0–50 µM) and the amount of cells invaded through Matrigel counted after 24 hours of incubation. As seen in Figure 2C, NAHA also inhibits invasion of MDA-MB-231 cells.

Cancer metastasis and invasiveness is associated with the expression of serine protease, urokinase-type plasminogen activator (uPA) which overexpression was reported in various human malignant cancers and was linked to advanced tumors and decreased survival time [17]. Therefore, we evaluated whether the inhibition of invasive behavior of breast cancer cells by NAHA could be mediated by the suppression of uPA secretion. MDA-MB-231 cells were treated with NAHA (0–50 µM) for 24 hours and the expression of uPA was determined in cell conditioned medium by Western blot analysis. As seen in Figure 2D, NAHA markedly reduced the secretion of uPA from MDA-MB-231 cells suggesting that NAHA inhibits invasiveness of breast cancer cells by the down-regulation of expression of uPA.

### Effect of NAHA on capillary morphogenesis of endothelial cells

Cancer progression and metastasis is also linked to the angiogenesis, which can be (on the cellular level) characterized by the tube formation (capillary morphogenesis) of endothelial cells. Indeed, we have recently demonstrated that highly invasive breast cancer cells MDA-MB-231 secrete vascular endothelial growth factor (VEGF) which stimulated capillary morphogenesis of endothelial cells [18]. To determine the effect of NAHA on the secretion of VEGF, MDA-MB-231 cells were treated with NAHA (0–50 µM) for 24 hours, cell culture media (conditioned media) collected and VEGF levels evaluated by ELISA as described in *Materials and Methods*. As seen in Figure 2E, NAHA markedly suppressed secretion of VEGF from MDA-MB-231 cells into media. Next, we determined whether NAHA suppresses capillary morphogenesis of human epithelial aortic cells (HAECs). Therefore, HAECs grown on Matrigel were treated with NAHA (0–50 µM) for 24 hours, and tube formation were evaluated. As seen in Figure 2F, NAHA suppressed capillary morphogenesis of HAEC cells in a dose-response manner. Collectively, our data suggest that NAHA inhibits capillary morphogenesis of endothelial cells and this effect is mediated through the suppression of secretion of VEGF from breast cancer cells.

### Effect of NAHA on tumor growth *in vivo*

**Figure 3:**
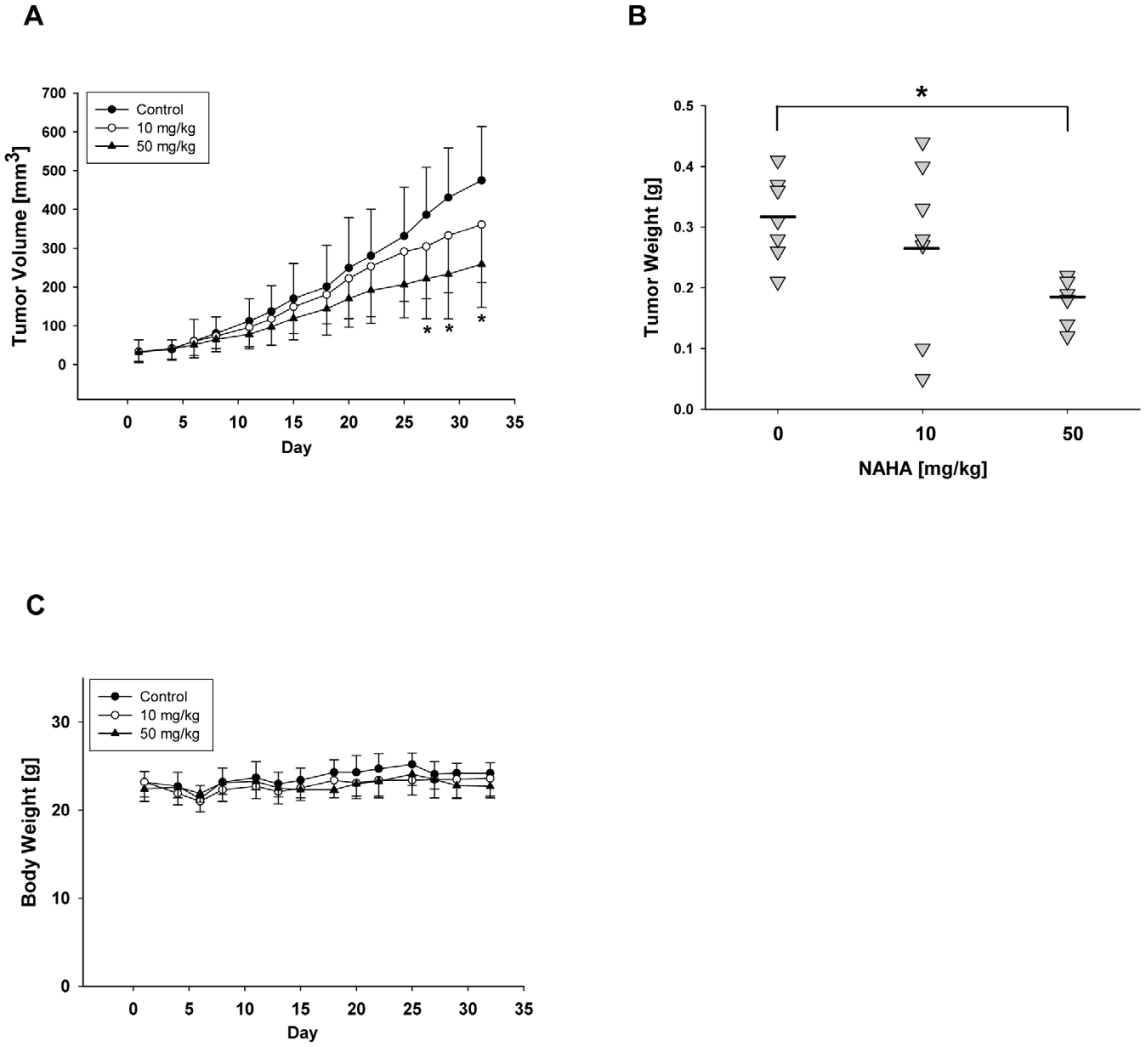
NAHA inhibits growth of breast cancer cells *in vivo*. (A) Tumor volume. MDA-MB-231 cells were inoculated s.c. in the right flank of each mouse. Seven days after inoculation, mice were randomly divided into 3 groups (n = 10) and treated with NAHA (i.p., 10 mg or 50 mg/kg of body weight/3 times per week) or vehicle (n = 11) for 32 days. Tumor volume was measured three times a week. Data are mean ± SD, * significantly different from control (p<0.05) using a two-sample Student t-test. (B) Tumor weight, * p<0.05. (C) Body weight of mice.

**Figure 4:**
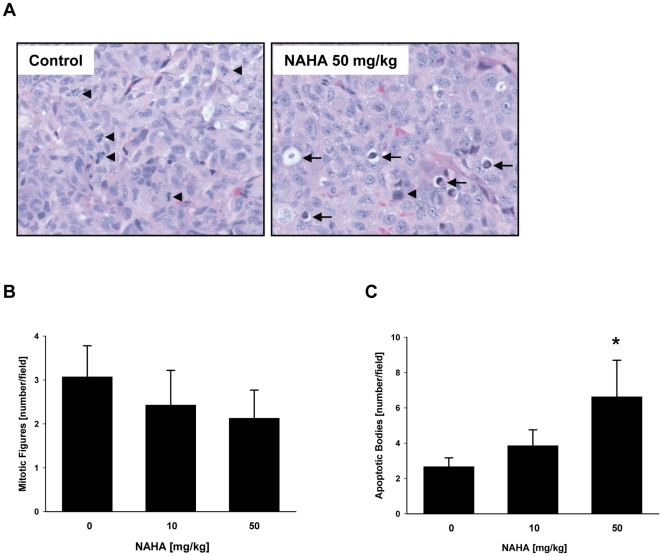
Histology of MDA-MB-231 xenografts. Tumors dissected from animals were sectioned and stained with (A) H&E, and (B) mitotic figures (arrowheads), and (C) apoptotic bodies (arrows) evaluated as described in *Materials and Methods*.

**Figure 5:**
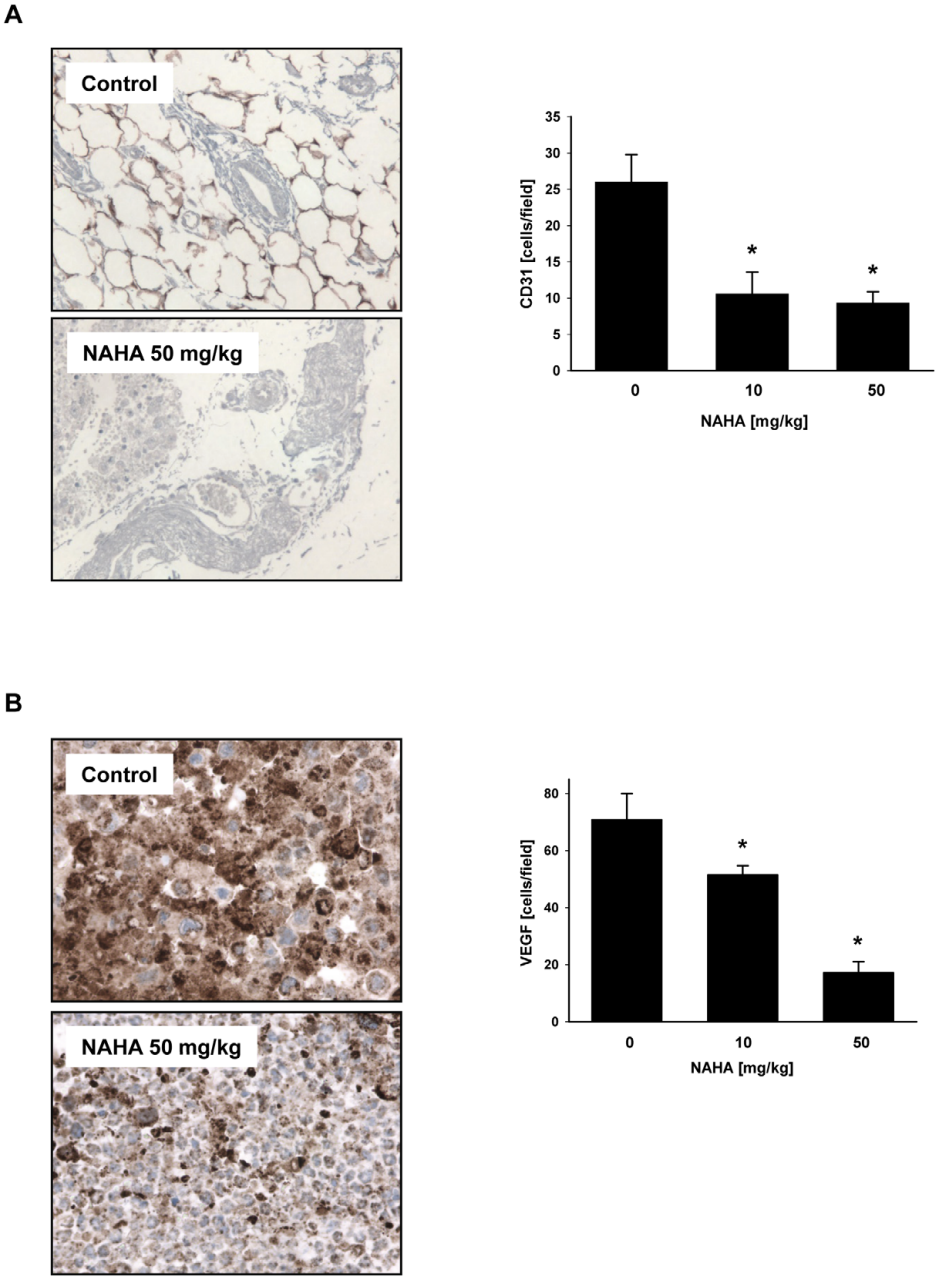
Expression of CD31 and VEGF in tumors. The expression of (A) CD31, and (B) VEGF were quantified as described in *Materials and Methods*. Data are mean ± SD (n = 6–10), * significantly different from control (p<0.05) by ANOVA.

To evaluate the effect of NAHA *in vivo* we employed a xenograft model of human breast cancer cells MDA-MB-231 subcutaneosly implanted into nude mice as described in *Materials and Methods*. Intraperitoneal administration of NAHA (50 mg NAHA/kg of body weight/3 times per week) for 32 days significantly suppressed tumor volume in nude mice (Figure 3A). In addition, NAHA treatment significantly suppressed final tumor weight (Figure 3B). Moreover, we did not observe any side effect of NAHA and there were no differences in body weight between control and treatment groups (Figure 3C), suggesting that NAHA is not toxic. In addition, we did not observe any pathological changes in the liver, spleen, kidney, lung and heart in NAHA treated animals. The H&E staining revealed that suppression of growth by NAHA is mediated by the slight inhibition of proliferation as demonstrated by the decreased mitotic figures (Figures 4A and B) and significant increase of apoptotic bodies in breast cancer xenografts (Figures 4A and C). In addition, NAHA also suppressed angiogenesis *in vivo* as demonstrated by significant decrease of CD31 (Figure 5A) and VEGF (Figure 5B) positive cells in tumors from animals treated with NAHA. In summary, NAHA suppresses growth of breast cancer xenografts through the inhibition of proliferation, induction of apoptosis and suppression of angiogenesis.

## Discussion

In the present study we evaluated the effect of newly synthesized derivate of hydroxamic acid, *2-[Benzyl-(2-nitro-benzenesulfonyl)-amino]-N-hydroxy-3-methyl-N-propyl-butyramide* (NAHA), on the growth, angiogenesis and invasive behavior of breast cancer cells. Here we show that NAHA markedly suppressed cancer cell proliferation (anchorage-dependent growth), cancer cell colony formation (anchorage-independent growth), invasive behavior of cancer cells (cell adhesion, migration and invasion) and capillary morphogenesis of endothelial cells. Moreover, we demonstrate that NAHA inhibits tumor growth and angiogenesis in xenograft model of human breast cancer.

Although NAHA inhibits proliferation of breast cancer as well as normal mammary cells, this effect is cytostatic because cell viability is affected only marginally in all tested cells. Moreover, the suppressive effect of NAHA on cell proliferation was prominent on the highly invasive breast cancer cells. Furthermore, the DNA microarray analysis revealed that NAHA down-regulates expression of several genes on mRNA levels, from which the down-regulation of expression of Cdk2 and CDC20 was also confirmed on the protein level. CDC20 is a protein regulating spindle checkpoint controlling the mitotic chromosome segregation during cell proliferation, which has been recently suggested as a potential therapeutic target [19], [20]. Indeed, the overexpression of CDC20 was demonstrated in breast cancer cell lines and primary breast tumors but not in normal mammary epithelial cells or breast tissues [19], [21], [22]. Therefore, NAHA suppresses growth of breast cancer cells through the down-regulation of expression of CDC20.

Cell invasion is the result of the activity of proteolytic enzymes such as MMPs, cysteine proteases, and uPA [23], [24], [25]. Because *N*-alkylation of hydroxamic acids eliminates their MMP inhibitory activity [6], [7], [8], our data suggest that *N*-alkylated NAHA inhibited cell invasion through the different mechanism, the suppression of secretion of uPA from breast cancer cells. In addition, we and others have previously reported that inhibition of uPA suppressed invasion of breast cancer cells [26], [27], [28]. Inhibition of cell adhesion and cell migration by NAHA can be mediated through the uPA-uPAR receptor signaling independently of uPA activity, because secreted uPA binds to uPAR and forms a complex with integrin receptor α_V_β_3_, which is ligated to vitronectin [29]. In addition, integrin receptor α_V_β_3_ is involved inf adhesion and migration of breast cancer cells [16]. Therefore, inhibition of uPA secretion by NAHA will suppress the formation of uPA-uPAR-α_V_β_3_-vitronectin complex, resulting in the inhibition of adhesion and motility of MDA-MB-231 cells.

Here, we show that NAHA suppresses secretion of VEGF from MDA-MB-231 and inhibits capillary morphogenesis (tube formation) of endothelial cells. We have previously demonstrated that the secretion of VEGF from MDA-MB-231 induces tube formation of vascular endothelial cells, whereas the inhibition of VEGF secretion suppresses this pro-angiogenic effect [18]. Moreover, suppression of endothelial capillary morphogenesis through the inhibition of secreted VEGF from a variety of cancer cells is well documented [30], [31], [32], [33]. In addition to the inhibition of cancer growth and invasive behavior of cancer cells, NAHA suppresses one of the first steps in angiogenesis – capillary morphogenesis of endothelial cells, and could therefore contribute to the inhibition of tumor angiogenesis.

In addition to its *in vitro* activity, NAHA suppresses tumor growth in a xenograft model of human breast cancer cells and this effect is associated with decreased proliferation and induced apoptosis of cancer cells. Moreover, NAHA also suppresses angiogenesis of breast tumors and down-regulates expression of VEGF in tumors *in vivo*.

In conclusion, NAHA, the *N*-alkyl derivate of hydroxamic acid, is a novel promising anti-cancer and anti-angiogenic compound. Therefore, it is possible to expect that the compounds based on the structure of NAHA will not exhibit detrimental effects of the previous hydroxamate-based MMP inhibitors. Further studies are warranted to synthesize more potent NAHA analogs, to develop specific targeted delivery to cancer cells, and to evaluate their effects on breast cancer growth and metastasis *in vivo*.

## Materials and Methods

### Reagents

Synthesis and analysis of *2-[Benzyl-(2-nitro-benzenesulfonyl)-amino]-N-hydroxy-3-methyl-N-propyl-butyramide* (NAHA) were previously described [9]. DMSO was purchased from Sigma (St. Louis, MO, USA). NAHA was dissolved in DMSO at a concentration of 50 mM and stored at −20°C.

### Cell culture

Primary human mammary epithelial cells (HMECs) were obtained from Lonza (Walkersville, MD) and grown in Mammary Epithelial Cell Growth Medium (MEGM, Lonza) with supplements according to manufacturer's protocol. Non-tumorigenic MCF-10A human mammary epithelial cells and human breast cancer cells MCF-7 and MDA-MB-231 were obtained from ATCC (Manassas, VA). MCF-10A cells were maintained in DMEM/F12 medium containing 5% horse serum (HS), insulin (10 µg/ml), epidermal growth factor (EGF, 20 ng/ml), cholera toxin (100 µg/ml), hydrocortisone (0.5 µg/ml), penicillin (50 U/ml), and streptomycin (50 U/ml). MCF-7 and MDA-MB-231 cells were maintained in DMEM medium containing penicillin (50 U/ml), streptomycin (50 U/ml), and 10% fetal bovine serum (FBS). Media and supplements came from Invitrogen (Grand Island, NY, USA). HS and FBS were obtained from Hyclone (Logan, UT, USA). Human aortic endothelial cells (HAECs) were obtained from Clonetics (Walkersville, MD, USA) and maintained in MEBM medium supplemented with EGM®-2 MV SingleQuots kit and 1% antibiotic-antimycotic solution (100×). HAECs were maintained at 37°C in a humidified atmosphere in the presence of 5% CO2. The cells were not used for more than 12 passages in our experiments. MEBM complete medium, and EGM®-2 MV SingleQuots kits were purchased from Clonetics.

### Cell proliferation, cell viability and colony formation

Proliferation and cell viability of HMECs, MCF-10A, MCF-7 and MDA-MB-231 cells treated with NAHA (0–50 µM) for 24 hours was determined as we previously described [17], [18]. Colony formation of MDA-MB-231 cells incubated in the presence of NAHA (0–50 µM) was evaluated as described [34]. Data points represent mean ± SD in one experiment repeated at least twice.

### DNA microarrays

MDA-MB-231 cells were treated with NAHA (0, 25, 50 µM) for 24 hours and total RNA isolated with RNAeasy (Qiagen, Valencia, CA, USA). This RNA was used for the evaluation of cell cycle regulatory genes with Cell Cycle Oligo GEArray according to the manufacturer's protocol (SA Biosciences, Frederick, MD, USA).

### Western blot analysis

MDA-MB-231 cells were treated with NAHA (0–50 µM) for 24 hours and whole cell extracts prepared as previously described [35]. Equal amounts of proteins (20 µg/lane) were separated on NuPAGE 4–12% Bis-Tris gel (Invitrogen, Carlsbad, CA, USA) and transferred to a PVDF membrane (Millipore, Bedford, MA, USA). The protein expression was detected with the corresponding primary antibodies: anti-CDC20, anti-Cdk2, anti-cyclin-B1, anti-PCNA, and anti-β-actin, (Santa Cruz Biotechnology, Santa Cruz, CA, USA). Protein expression was visualized using the ECL Western Blotting Detection System (Amersham Biosciences, Buckinghamshire, UK).

### Cell invasiveness

Cell adhesion of MDA-MB-231 cells treated with NAHA (0–50 µM) for 24 hours was performed, as we described [36]. Cell migration of MDA-MB-231 cells treated with NAHA (0–50 µM) for 5 hours was evaluated, as previously described [35], [36]. Cell invasion of MDA-MB-231 cells treated with NAHA (0–50 µM) for 24 hours was performed as we described [36]. Data points represent the mean ± SD of three individual filters within one representative experiment repeated at least twice.

### uPA secretion

uPA secretion from MDA-MB-231 cells treated with NAHA (0–50 µM) for 24 hours was evaluated as we previously described [35]. The loading was normalized to the same amount of treated cells.

### Secretion of VEGF

MDA-MB-231 cells were treated with NAHA (0–50 µM) for 24 hours, cell media collected and secretion of VEGF was determined by ELISA kit (R&D Systems, Minneapolis, MN, USA).

### *In vitro* endothelial cell morphogenesis assay (capillary morphogenesis)

Human aortic endothelial cell (HAEC) differentiation into capillary-like structures was examined using a two dimensional Matrigel-based assay as previously described [18]. Initially, 200 µl of ice cold growth factor reduced-Matrigel (Becton-Dickson Labware, Bedford, MA, USA) was placed into each well of a 24-well plate. HAECs were incubated with NAHA (0–50 µM) for 16 hours in MEBM media supplemented with EGM®-2 MV SingleQuots kit. HAECs differentiated into capillary-like structures within 16 hours of incubation at 37°C. After 16 hours of incubation, the structures were evaluated microscopically (10×) by inverted Olympus CK40 microscope as previously described [18]. Quantification of the capillary-like structures was performed by counting the number of nodes/field, where node is defined as an intersection of at least three cells. Each sample was assayed in triplicate and repeated at least three times.

### Human breast cancer xenograft experiments

Female nude immunodeficient mice (nu/nu), 6 weeks old, were purchased from Harlan (Indianapolis, IN, USA) and housed in accordance with protocol approved by the Institutional Laboratory Animal Care and Use Committee of the Methodist Research Institute. On day 0, human breast cancer MDA-MB-231 cells (1×10^6^) suspended in 200 µL of DMEM medium were inoculated subcutaneously (s.c.) in the right flank of each mouse. Seven days after inoculation, mice were randomly divided into three groups (n = 10) and treated 3 times per week with 100 µl intraperitoneal (i.p.) injection of vehicle (control) or NAHA (10 mg/kg and 50 mg/kg). Tumor sizes were measured three times a week using calipers and their volumes were calculated using a standard formula: tumor volume (mm^3^) = (L×W^2^)×1/2, where L is the length and W is the width of the tumor. The animals were sacrificed after 32 days. Body weight was measured three times weekly. The protocol for animal experiments was approved by the Animal Research Committee at the Methodist Hospital (protocol number 2008-33) according to the NIH guidelines for the Care and Use of Laboratory Animals.

### Histology and Immunohistochemistry

Tumors were harvested, fixed in 10% neutral buffered formalin at 4°C for 24 hours followed tissue processing overnight, and then embedded in paraffin. Five-micrometer sections were stained for routine H&E. The slides were viewed under a Leica microscope (Leica Instruments, Melville, NY, USA) and the number of cells in the viable tumor cell area was quantitated by counting both apoptotic bodies and mitotic figures in four 20× fields of view for *n* = 10 tumors per group. Rat anti-mouse CD31 monoclonal antibody (clone SZ31) from Dianova (Hamburg, Germany) and anti-VEGF antibody were utilized using standard automated immunohistochemical techniques on the DAKO Autostainer and 3,3′-diaminobenzadine as chromagen. For the CD31 positive staining endothelial cells in small vessels, the small capillaries and small vessels were counted in one 16× field. The CD31 and VEGF positive cells were quantified by Image J (National Institutes of Health, Bethesda, MD, USA). Three fields were counted and averaged. The large necrotic areas in the xenograft were excluded from analysis and only viable tumor areas were used. The area captured averaged about 15 to 25% of the whole cross sectional area of the tumor. For the immunohistochemical quantification QC, three randomly selected images (16× power fields) each (total area, 7.3 mm^2^) from the tumor periphery were analyzed in each tumor per group. Tumor periphery indicates a region less than one field of view from the tumor edge at ×200 magnification (<1,360 microns), and tumor core indicates areas >1,360 microns from the tumor edge.

### Statistical analysis

Data are expressed as the mean ± standard deviation (SD). Statistical comparison between groups of data was carried out using ANOVA. P<0.05 was considered to be significant.
